# Concurrent Aspirin Use Is Associated with Improved Outcome in Rectal Cancer Patients Who Undergo Chemoradiation Therapy

**DOI:** 10.3390/cancers13020205

**Published:** 2021-01-08

**Authors:** Mark K. Farrugia, Mark D. Long, David M. Mattson, Leayn T. Flaherty, Bowen Dong, Eduardo Cortes Gomez, Lei Wei, Agnieszka K. Witkiewicz, Song Yao, Pawel Kalinski, Anurag K. Singh

**Affiliations:** 1 Department of Radiation Medicine, Roswell Park Cancer Institute, Elm and Carlton Streets, Buffalo, NY 14263, USA; mark.farrugia@roswellpark.org (M.K.F.); david.mattson@roswellpark.org (D.M.M.); Leayn.Flaherty@RoswellPark.org (L.T.F.); 2 Department of Biostatistics & Bioinformatics, Roswell Park Cancer Institute, Buffalo, NY 14263, USA; Mark.Long@RoswellPark.org (M.D.L.); Eduardo.CortesGomez@RoswellPark.org (E.C.G.); Lei.Wei@RoswellPark.org (L.W.); 3 Departments of Medicine and Center for Immunotherapy, Roswell Park Cancer Institute, 945 CSC Building, Elm & Carlton Streets, Buffalo, NY 14263, USA; Bowen.Dong@RoswellPark.org; 4 Center for Personalized Medicine, Roswell Park Cancer Institute, Buffalo, NY 14263, USA; Agnieszka.Witkiewicz@RoswellPark.org; 5 Department of Cancer Prevention and Control, Roswell Park Cancer Institute, Buffalo, NY 14263, USA; Song.Yao@RoswellPark.org; 6 Jacobs School of Medicine and Biomedical Sciences, University at Buffalo, Buffalo, NY 14263, USA

**Keywords:** rectal cancer, chemoradiation, aspirin, tumor response, survival

## Abstract

**Simple Summary:**

Those with a history of colorectal cancer benefit from aspirin following completion of treatment, however, it is believed that this benefit is restricted to patients who have tumors with certain mutations. It is not known whether taking aspirin during active treatment helps patients or whether it depends on tumor genetics in this setting. We investigated 147 patients who underwent chemoradiation for rectal cancer at our institution, including 42 who were taking aspirin at the time of chemoradiation. Concurrent aspirin use was associated with significantly improved tumor control and survival. Genetic analysis of the tumor did not indicate that these benefits were restricted to certain molecular conditions. Interestingly, aspirin was associated with more advantageous immune response in tumor specimens. Based on these findings, the addition of aspirin to chemoradiation should be considered for rectal cancer patients, independent of molecular conditions.

**Abstract:**

Background: The benefit of aspirin in rectal cancer during chemoradiation therapy (CRT) and the factors affecting its efficacy are not well characterized. We compared the outcomes of rectal patients undergoing neoadjuvant CRT based on aspirin use. Methods: Patients undergoing CRT for rectal cancer from 2010 to 2018 were evaluated. Aspirin use was determined by medication list prior to treatment. RNA sequencing and subsequent gene set enrichment analysis was performed on surgically resected specimens. Results: 147 patients underwent neoadjuvant CRT with a median follow-up of 38.2 months. Forty-two patients were taking aspirin prior to CRT. Aspirin users had significantly less local and distant progression, and improved progression-free and overall survival. On RNA-sequencing, neither *PI3KCA* nor *KRAS* mutational status were associated with the benefit of aspirin use or tumor downstaging. PTGS2/COX2 expression trended lower in aspirin users, but not with tumor response. Aspirin use was associated with increases of M1 macrophages, plasma cells, CD8+ T cells, and reduction of M2 macrophages in the resected tumor. Conclusions: Concurrent aspirin use during neoadjuvant CRT was associated with improved local and distant tumor control leading to significantly improved survival. Neither mutations in *KRAS* or *PI3CKA*, nor the levels of COX-2 expression at the time of resection of the residual tumor were predictive of these aspirin benefits.

## 1. Introduction

Aspirin is an irreversible inhibitor of cyclooxygenase 1 and 2 (COX-1 and COX-2) with a variety of clinical uses including pain relief, anticoagulation, and chemoprevention. Numerous epidemiologic studies have demonstrated that aspirin use can reduce the incidence of colorectal cancer (CRC) and colorectal adenomas, ostensibly through inhibition of COX-2 mediated progression of precursor lesions [1,2,3,4,5,6,7,8]. Inspired by its efficacy as a chemo-preventive agent, several groups observed improvements in both cause-specific survival and overall survival with adjuvant aspirin use following a CRC surgery [9,10,11,12]. Interestingly, several reports have suggested that the oncologic benefit of adjuvant aspirin is restricted to those with PI3KCA-mutated CRCs [13,14]. 

In contrast to preventative and adjuvant use, less attention has been given to neoadjuvant aspirin therapy for CRC [4]. The only published prospective study found significantly improved tumor downstaging, pathological response, progression-free survival (PFS), overall survival (OS), and lower rate of distant metastases associated with neoadjuvant aspirin use [15]. While there was no significant difference in local failure rates, those taking aspirin had a lower risk of metastatic failure. In contrast to these findings, three retrospective studies of CRC patients observed no significant benefits of aspirin to tumor response or survival [16,17,18]. As such, the benefit for the addition of aspirin to neoadjuvant therapy in CRC patients is controversial, and the predictive value of biomarkers, such as PIK3CA mutations, has not been studied. 

In the current study, we investigated the impact of aspirin use during neoadjuvant chemoradiation therapy (CRT) for rectal cancer on treatment response, tumor control, as well as survival-based outcomes and evaluated genomic and molecular biomarkers as potential predictors for treatment response. 

## 2. Materials and Methods

### 2.1. Patients

We assembled a cohort of 182 patients who underwent radiotherapy for rectal cancer at our institution from 2010 to 2018. Of these patients, we excluded those who did not receive at least 45 Gy, were treated with palliative or adjuvant intent, or had a non-adenocarcinoma histology, resulting in a sample size of 147 patients in the final analysis (Figure S1). This analysis was approved by the Roswell Park Comprehensive Cancer Center Institutional Review Board (BDR 105718, BDR 114619) for human subject protection. 

### 2.2. Patient Evaluation and Treatment

All patients were initially evaluated by a surgical oncologist. Standard work up included endoscopy and computed tomography of the chest, abdomen, and pelvis, and magnetic resonance imaging of the rectum. All patients had histologically confirmed diseases. Staging was completed using American Joint Committee on Cancer (AJCC) 7th edition. Cases were presented to a multidisciplinary committee where a consensus recommendation was formulated. In agreement with National Comprehensive Cancer Center guidelines, patients with metastatic diseases isolated to the liver were candidates for definitive treatment if appropriate [19]. The range of prescribed radiation doses were 45–54 Gy, with over 90% of patients receiving 50.4 Gy in 1.8 Gy fractions. Most patients were treated with three dimensional conformal radiation therapy. Nearly all patients received concurrent chemotherapy, generally capecitabine. Induction therapy was composed of capecitabine or 5-flurouracil, leucovorin, and oxaliplatin, and recommended based on multidisciplinary consensus. At approximately 6 weeks following completion of treatment, patients were reassessed by a surgical oncologist for potential resection. If applicable, patients were offered the option to defer surgical resection and followed per the “watch and wait” approach [20]. In some instances, patients were recommended surgery, but refused. In this scenario, patients would receive adjuvant chemotherapy if they did not undergo prior induction chemotherapy. 

### 2.3. Patient Demographics and Comorbidities

Use of medications, including aspirin, β-blockers, calcium channel blockers, statins, angiotensin converting enzyme (ACE) inhibitors/angiotensin receptor blocker (ARBS), and non-steroidal anti-inflammatory drugs were recorded using the medication list at the time of initial consultation. Tobacco use was determined at the time of consultation [21]. Patients who were still actively smoking at this time were considered current smokers. The location of the tumor was documented relative to anal verge. For the location of progression, local was defined as progression within the initial radiation treatment fields. 

### 2.4. Tumor RNA-Sequencing and Bioinformatic Processing

Formalin-fixed, paraffin-embedded (FFPE) tumor specimens from 21 patients were available from Roswell Park Pathology Network Shared Resource (PNSR) and subjected for RNA extraction. SureSelectXT RNA Direct (Agilent Technologies, Santa Clara, CA, USA) was used for library preparation, and 100 base paired-end sequencing performed on a NovaSeq6000 (Illumina Inc., San Diego, CA, USA) following manufacturer’s recommended protocols at the Roswell Park Genomics Shared Resource. Paired-end reads were assessed for quality control using fastqc (v0.10.1) and mapped to GRCh38.p7 human reference genome and GENCODE (v25) annotation database using STAR (v2.7.0f) [22]. Raw feature counts were normalized, and differential expression analyses performed using DESeq2 [23]. Differential expression rank order was utilized for subsequent Gene Set Enrichment Analysis (GSEA), performed using the clusterProfiler package in R. Cell type estimation analysis was performed on normalized expression values using xCell [24]. Somatic mutations in selected cancer hotspots were identified from RNA-Seq data using a customized script as previously described [25]. The following cancer hotspots were included in the analysis: *KRAS* G12, *BRAF* V600, and *PIK3CA* (E542 and E545 in exon 9, and H1047 in exon 2) [26]. All identified mutations were manually inspected to exclude potential false positive due to sequencing or mapping artifacts.

### 2.5. Statistical Analysis 

Pearson’s chi square test, Fisher’s exact test, and Wilcoxon tests were used to examine differences between groups. Local failure was defined as progression within the initial radiation treatment fields, whereas distant progression was defined as outside of the initial radiation treatment fields. Differences in treatment failure and location were determined by Pearson’s chi square test. Progression-free survival (PFS) was defined as the date of first radiation treatment to date of local or distant progression as defined by progression on imaging or biopsy. Patients without progression or death without history of relapse were censored at the last contact. Overall survival (OS) was defined as the date of first treatment to the date of death due to any causes. Kaplan-Meier survival curves were generated using R version 4.0.2 with package survival version 3.2–7. To adjust for potential confounding, covariates with a *p*-value of <0.1 in univariate test were included in a multivariate Cox regression models. Propensity score matching for age (<61 years, 61 years or more), sex, Karnofsky Performance Status (KPS) (100–80, <80), T stage (T1–2, T3–4), node positivity (N0, N+), isolated metastatic to liver, receipt of induction chemotherapy, and surgery was performed using a 1:1 ratio and nearest neighbor method, caliper length of 0.1 in R with package MatchIt version 3.0.2 [27]. 

## 3. Results

A total of 147 patients underwent neoadjuvant CRT for rectal cancer. Patients’ demographic characteristics are summarized in Table 1. The median age was 61.0 years (interquartile range (IQR) 52–73 years); the majority of the patients were male (*n* = 93, 63.3%); had KPS of 80–100 (*n* = 139, 94.6%), with lesions at least 10 cm from the anal verge (*n* = 75, 52.8%). The majority of patients had T3 tumors (*n* = 123, 84.8%), N1 disease (*n* = 65, 45.5%), and 22 (15.0%) patients had documented isolated liver involvement at diagnosis. Regarding treatment, 55 (37.4%) patients received induction chemotherapy, 143 (98.0%) patients had concurrent chemotherapy with radiation, while 75 (51.4%) patients underwent surgical resection for their rectal tumors. Most patients were non-smokers (*n* = 73, 50.3%). The median follow-up time was 38.2 months (IQR 24.6–59.1 months), during which 61 (41.5%) disease progression events and 44 (29.9%) deaths were documented. 

At the time of diagnosis, 42 (28.6%) patients were taking aspirin, including daily aspirin doses of 81 mg in 37 (88.1%) patients, 325 mg in 2 (4.8%) patients, and dosages not documented in 3 (7.1%) patients. Aspirin users were more likely to be male (78.6% vs. 57.1%, *p* = 0.015) and no other differences in patient demographic or clinicopathologic variables were noted. 

In comparison to non-aspirin users (*n* = 105), aspirin users (*n* = 42) had less local (9.5% vs. 14.3%) and distant progression (14.3% vs. 34.3%) (*p* = 0.019). Of the 75 patients who underwent resection following neoadjuvant CRT, 22 (52.4%) were aspirin-users, and 53 (50.5%) were non-users. In comparing treatment response of these groups, pathologic complete response (pCR) was observed in 6 (27.2%) vs. 6 (11.3%), partial response in 9 (40.9%) vs. 29 (54.7%), and no response/progression in 7 (31.8%) vs. 18 (34.0%) for aspirin users vs. non-users, respectively (*p* = 0.21) (Table 2).

**Figure 1:**
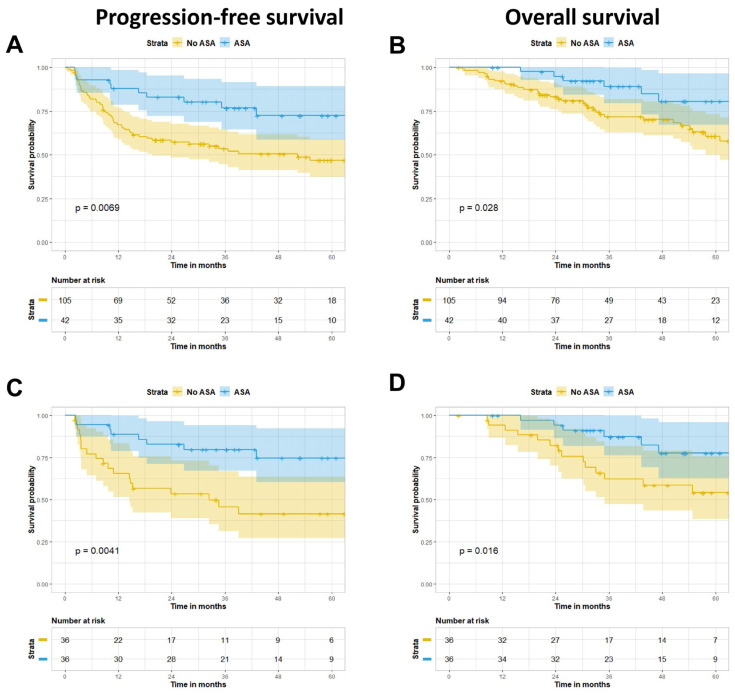
Kaplan-Meier survival estimates and 95% confidence intervals in the unmatched (*n* = 147) and matched (*n* = 72) cohorts for relapse-free survival (**A**,**C**) and overall survival (**B**,**D**), respectively.

In univariate Cox regression analysis, significant clinical variables associated with PFS included isolated involvement of the liver (Hazard Ratio (HR) = 2.6, 95% confidence interval (CI) 1.4–4.7, *p* = 0.002); surgical resection (HR = 0.36, 95% CI 0.21–0.61, *p* ≤ 0.0001); induction chemotherapy (HR = 1.8, 95% CI 1.1–3.0, *p* = 0.02); and aspirin use (HR = 0.4, 95% CI 0.21–0.8 95% CI, *p* = 0.009) (Table 3). Moreover, KPS (HR = 2.5, 95% CI 0.88–6.9, *p* = 0.086), isolated involvement of the liver (HR = 2.3, 95% CI 1.2–4.4, *p* = 0.016), surgical resection (HR = 0.2, 95% CI 0.1–0.41), p ≤ 0.001), and aspirin use (HR = 0.4, 95% CI 0.19–0.93, *p* = 0.033) were associated with OS. In multivariable Cox regression analysis, taking aspirin during neoadjuvant CRT remained significantly associated with both PFS (HR = 0.35, 95% CI 0.17–0.7, *p* = 0.003) and OS (HR = 0.36, 95% CI, 0.16–0.82, *p* = 0.015), after adjusting for isolated liver involvement and surgical resection in the models. In support of these findings, 36 propensity score match pairs were generated for aspirin users and non-users. All variables were well balanced (Table S1). In the unmatched cohort, aspirin use was associated with improved PFS (*p* = 0.007) and OS (*p* = 0.028) on Kaplan-Meier survival estimates, and these observations held on matching (PFS; *p* = 0.004 and OS; *p* = 0.02) (Figure 1). The estimated 3-year PFS and OS for the unmatched cohorts were 64.5% vs. 41.3% and 79.3% vs. 62.7% for aspirin users vs. non-users, respectively, whereas PFS and OS were 67.3% vs. 31.5% and 76.4% vs. 44.4% in the matched cohorts, respectively. 

To explore molecular and genomic features of rectal tumors that might be related to the better outcomes observed in patients taking aspirin, primary tumor tissues from 21 patients (8 aspirin users, 13 non-users; 9 responders, and 12 non-responders) who had archived samples procured by Roswell Park Pathology Network Shared Resource (PNSR) were profiled using RNA sequencing. Demographic characteristics of this patient population are shown in Table S2. Hotspot mutations in three candidate genes that were previously implicated in response to aspirin in colorectal cancer patients, including *PIK3CA*, *KRAS*, and *BRAF*, were interrogated and summarized in Table S3. A tumor sample from one patient, who harbored an E545D mutation in *PIK3CA*, showed partial pathological response to CRT, but the patient did not take aspirin. Tumors from two other patients harbored a mutation in *KRAS*, both of whom had a response to CRT, and one took aspirin. No *BRAF* mutations were found in this patient population. 

**Figure 2:**
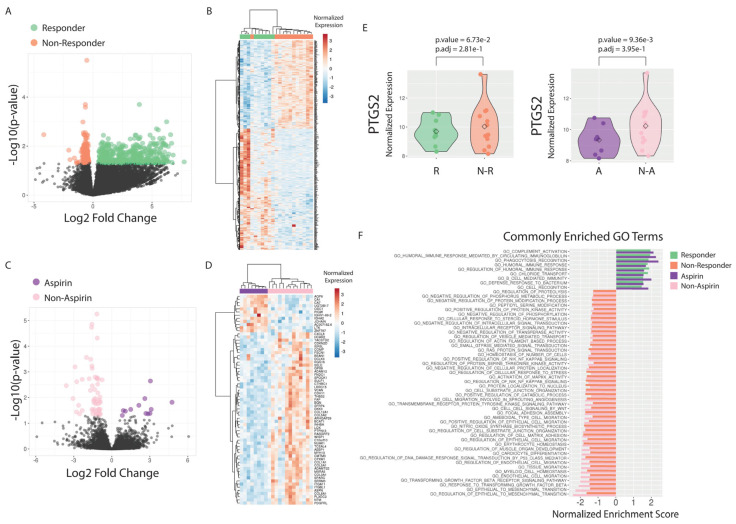
Differential expression determined by RNA-seq in chemoradiation therapy (CRT) responders (*n* = 9) relative to non-responders (*n* = 12) and aspirin users (*n* = 8) relative to non-users (*n* = 13). (**A**) Volcano plot depicting differential expression analysis between CRT responders and non-responders. Genes with significant difference between groups are highlighted (BH adjusted *p*-value < 0.05; responders = green, non-responders = orange). (**B**) Heatmap and supervised clustering of samples based on genes associated with CRT response. (**C**) Volcano plot depicting differential expression analysis between aspirin users and non-users. Genes with significant difference between groups are highlighted (BH adjusted *p*-value < 0.05; aspirin users = purple, non-users = pink). (**D**) Heatmap and supervised clustering of samples based on genes associated with aspirin use. (**E**) Normalized PTGS2 expression in patients associated with each comparison. (**F**) GO terms significantly associating with both CRT response and aspirin use.

Differential expression (DE) analysis comparing transcriptomes of CRT responders to non-responders revealed broad significant (Benjamini-Hochberg (BH) adjusted *p*-value < 0.05) expression changes (678 DE genes; 435 upregulated in responders, 243 upregulated in non-responders) (Figure 2A,B, Table S4). Similar analysis comparing aspirin users to non-users revealed a smaller but significant DE gene program (67 DE genes; 12 upregulated in aspirin users, 55 upregulated in non-users) (Figure 2C,D, Table S5). Only a single gene, *DLCK3*, was significant in both comparisons. CRT responders had elevated expression of *DLCK3* relative to non-responders (log2FC = 2.78, BH adjusted *p*-value = 0.01); yet aspirin users had reduced expression relative to non-users (log2FC = −2.80, adjusted *p*-value = 0.04), making it an unlikely candidate mediating the better response among patients taking aspirin. Notably, *PTGS2*/*COX2* expression trended lower in aspirin users relative to non-users (Figure 2E, log2FC = −1.43, unadjusted *p*-value = 0.009; BH adjusted *p*-value = 0.39).

Gene set enrichment analysis (GSEA) was performed to query functional pathways enriched in CRT response and aspirin use independently (Figure S2A,B, Tables S6 and S7). Highly enriched pathways associated with responder status included upregulation of sensory reception and loss of SWI/SNF signaling, HDAC activity, and stemness (Figure S2C). Pathways upregulated with aspirin use were largely associated with immune activity, including type-1 adaptive and innate immunity (which are suppressed by the key COX2 product, prostaglandin E2 (PGE_2_) [28], as well as antigen processing and presentation (Figure 2F)). Aspirin suppressed pathways associated with epithelial to mesenchymal transition (EMT) and stemness, as well as pathways previously associated with advanced gastric cancer. Pathways common to CRT response and aspirin use included those associated with the activation of adaptive immunity and suppression of EMT and transforming growth factor-beta signaling (Figure 2F). Importantly, PI3K signaling pathways (e.g., HALLMARK_PI3K_AKT_MTOR_SIGNALING) were not among the top functional enrichments, and interestingly, there was evidence of negative enrichment of this pathway among CRT responders (*p*-value = 0.004, *q*-value = 0.02). Furthermore, pathway activity analysis by single sample GSEA (ssGSEA) did not identify significant differences in PI3K/AKT signaling between aspirin users that responded to CRT and those that did not, suggesting that PI3K/AKT signaling is not a defining characteristic of patients benefiting from aspirin use in this cohort (Figure S2E).

**Figure 3:**
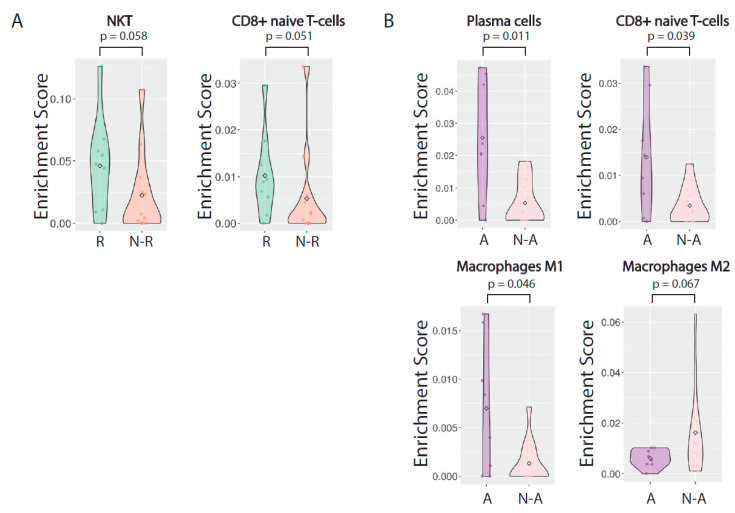
Assessment of the immune microenvironment associated with CRT response (*n* = 9 responders, *n* = 12 non-responders) and aspirin use (*n* = 8 aspirin users, *n* = 13 non-users). (**A**) Cell type enrichment analysis was performed on bulk expression profiles by xCell. Candidate immune populations showing significant (Wilcoxon rank sum test, *p* < 0.1) differences between CRT responders and non-responders and (**B**) between aspirin users and non-users are shown.

To infer the cellular compositions of bulk tissue samples, cell type enrichment analysis (xCell) was performed (Figure 3A,B, Tables S8 and S9). In concert with GSEA pathway enrichment patterns, cell type enrichment identified significantly (Wilcoxon rank sum test, *p* < 0.1) increased infiltration of plasma cells and M1 macrophages, with reduced M2 macrophages in aspirin users relative to non-users. Elevated proportions of CD8+ naïve T cells were observed in both CRT responders and aspirin users relative to non-responders and non-users, respectively. Natural killer T (NKT) cells were also elevated in CRT responders. These results suggest that aspirin use may impact the tumor immune microenvironment in a manner that impacts response to CRT.

## 4. Discussion

In the current study, aspirin users had numerically higher proportions of pCR following CRT, as well as significantly fewer local and distant failures. These findings translated to significantly improved PFS and OS with aspirin use on a multivariable model, and these observations remained significant after match pairing. Further investigation into molecular pathways impacting treatment response via RNA sequencing failed to show any association between alterations in PIK3CA signaling, aspirin use, and tumor downstaging.

Although aspirin can be used for the chemo-prevention of CRC, its use may increase the risk of bleeding [29]. This concern led to the current focus on the utility of aspirin in those already diagnosed with CRC, since the benefits in these patients may exceed that of the general population [8,9,12]. For adjuvant treatment, population-based studies observed significant improvements in cause-specific survival and overall survival with aspirin use [9,12]. In comparison, only a few studies have examined aspirin in the neoadjuvant setting, with conflicting results. Three retrospective studies reported no pathological or survival benefit, yet the numbers of patients on aspirin were fairly small [16,17,18]. A prospective analysis of aspirin use in CRC patients undergoing neoadjuvant chemoradiation therapy (CRT) found, compared to non-aspirin users (*n* = 204), patients taking aspirin (*n* = 37) had a significantly lower rate of distant metastases and significantly improved tumor downstaging, pathological response, 5-year PFS and OS; these findings concur with the current study [15]. These concordances increase confidence in the clinical benefits of aspirin use in CRC patients undergoing neoadjuvant therapy. 

Prostaglandins play an important role in colorectal carcinogenesis. As aspirin irreversibly inhibits the activity of COX-2, a rate limiting enzyme in the synthesis of prostaglandin E2 (PGE2), and activation of PI3K-AKT pathway upregulates COX2 expression, it was hypothesized that constitutional activation of this signaling pathway by PIK3CA mutations might be predictive of aspirin’s benefits for CRC patients. Data from the Nurses’ Health Study and the Health Professionals Follow-up Study support this, which found the benefit of adjuvant aspirin to be confined to those with mutated *PIK3CA* (cause-specific survival, HR 0.18, 95% CI 0.06–0.61), and no benefit was detected in patients with *PIK3CA*-wild type tumors (HR 0.96, 95% CI 0.69–1.32) [13]. In agreement, several other studies reported similar findings regarding the predictive value of *PIK3CA* mutations for the benefits of adjuvant aspirin use in CRC patients, as concluded in two separate meta-analyses to synthesize these results [14,30].

However, not all published studies support the importance of *PIK3CA* mutations to aspirin use in CRC patients. In a population-based cohort study of 740 CRC patients, Gray et al. did not find *PIK3CA* mutations to be predictive for aspirin benefit [31]. Similarly, in a large cohort of 1487 CRC patients by Kothari et al., *PIK3CA*-mutations did not significantly correlate with cancer-specific survival or overall survival in aspirin users [32]. Since *PIK3CA* mutations occur only in 10–15% of CRC patients, it was postulated that *PIK3CA*-mutations may be too restrictive, and this subpopulation alone cannot explain the benefits of aspirin on a greater proportion of CRC patients [33]. COX-2 expression has been evaluated as another biomarker for aspirin benefits, which was supported by the aforementioned study by Gray et al. [31]. Nevertheless, Reimers et al. found that neither COX-2 expression nor *PI3KCA* status were informative regarding aspirin use and patient outcome [34]. In addition, the potential predictive value of *KRAS* and *BRAF* mutations for aspirin benefits have also been investigated in CRC patients, yet the findings are still inconsistent across studies [35,36,37]. 

To our knowledge, no previous studies have examined PIK3CA mutations or other biomarkers in relation to aspirin benefits in CRC patients receiving neoadjuvant CRT. Based on RNA-seq data from a subset of our rectal cancer patient population, neither *PIK3CA* or *KRAS* mutational status, nor COX-2 mRNA expression were associated with the benefit of aspirin use or tumor downstaging. Furthermore, GSEA results also did not support the importance of the PI3K/AKT signaling pathway in response to aspirin use, yet this pathway appeared to be downregulated in patients who responded to CRT. Interestingly, aspirin use was associated with decreased COX-2 expression (Figure 2E) despite being an inhibitor of COX-2 activity, not its transcription or translation. This result can be explained by the existence of a unique positive feedback between the production of PGE_2_ and COX2 (the rate limiting enzyme in PGE_2_ production) [38,39]. Although the sample size of the patients included in genomic profiling is limited and only one patient was identified with *PIK3CA* mutations and two with *KRAS* mutations, our results, at least, are not directionally consistent with any significant predictive role of these mutations. 

Despite the negative results of the *PIK3CA* mutations and the PI3K signaling pathway, in the analysis of cell composition using bulk tissue RNA-seq data, we found a stronger infiltration of CD8+ naïve T cells to the tumor microenvironment among responders vs. non-responders to CRT, as well as among aspirin vs. non-aspirin users. Although the differences became non-significant after adjusting for multiple testing, likely due to limited sample size, the findings are in-line with the role of COX-2 in lowering the production of chemokines known to attract naïve, effector, and memory cytotoxic T lymphocytes (CTLs) and the activation and proliferation of T cells. This may explain the increased presence of CD8+ naïve T cells in the tumor microenvironment when COX-2 activity was inhibited by aspirin, which may, in turn, lead to a better response to CRT. These finding are consistent with previous data demonstrating the importance of the PGE_2_/COX2 system from tumors’ escape from immunosurveillance [38,39,40,41] These data, although preliminary, suggest a potential immunomodulatory mechanism underlying the clinical benefits of aspirin in CRC cancer patients and may warrant further investigation. 

Interestingly, while local control was improved with concurrent aspirin, there was a far larger impact on distal failure. This is in agreement with prior work which demonstrated a significant reduction in the development of metastatic disease with neoadjuvant aspirin use [17]. While modern treatment paradigms of locally advanced CRC have excellent rates of local control, distal failure remains problematic with approximately 20–30% of patients developing metastatic disease [42]. Two recent clinical trials, Rectal Cancer And Pre-operative Induction Therapy followed by Dedicated Operation (RAPIDO) and Organ Preservation in Rectal Adenocarcinoma (OPRA) have adopted a total neoadjuvant therapy approach to address this problem, with the added benefit of improved tumor downstaging, sphincter preservation, and in some instances, the avoidance of surgery [20,43,44]. The inclusion of prophylactic dose aspirin in neoadjuvant treatment is a cost-effective method to further improve the above disease metrics. Moreover, prophylactic dose aspirin is very well tolerated and associated with a minimal risk of bleeding [29]. 

In a small, randomized clinical trial, Haldar et al. reported a trend towards improved distant failure and 3-year recurrence rates, as well as a more favorable gene expression profile in CRC patients using perioperative etodolac for 20 days in conjunction with propranolol [45]. While not studying aspirin directly, Haldar et al. demonstrated that even short-term inhibition of prostaglandin synthesis can derive clinical benefit in CRC patients [45]. Although studies reporting the benefits of neoadjuvant or adjuvant aspirin are in long-term users, the optimal duration of aspirin treatment is not clear [9,10,11,12,13,14,15,31,34]. 

Currently, the National Comprehensive Cancer Network (NCCN) has made no recommendations regarding the adjuvant use of aspirin with respect to tumor genetic profile and states one can consider aspirin 325 mg for secondary prevention [19]. Aspirin use during neoadjuvant treatment has not been addressed. Based on the findings from our study and the existing literature, we recommend prophylactic dose aspirin in all CRC patients during CRT regardless of molecular profile without contraindications to treatment, given the low potential for harm and possibly significant benefit. We plan to follow these patients in a prospective cohort study to understand the long-term outcomes.

Beside a limited sample size in RNA-sequencing, another limitation of this study came from the use of post-treatment surgical specimens. Initial pre-treatment biopsies often performed at outside facilities were not available for genomic analysis, thereby precluding an investigation of tumors that responded completely to neoadjuvant treatment. Nevertheless, prior work investigating the tumor context dependency of aspirin often used surgical specimens from patients, many of whom had undergone neoadjuvant treatment; thus, this is not a limitation unique to our study. Further, post-treatment tumor specimens were appropriate for the analysis of genomic changes between responders and non-responders to CRT, and between aspirin and non-aspirin users. This data was limited to a single institution without external validation, lacked randomization data, and contained some heterogeneities in patient demographics and treatment within this cohort. However, except for gender, these factors were not significantly different between aspirin and non-aspirin users. Statistical models were employed to adjust for potential confounding effects. Clinical trials with randomization on key stratifying factors will be required to definitively confirm our results and allow a more direct, in depth analysis of the effects of CRT and aspirin on CRC tumors and their microenvironment. 

## 5. Conclusions

Our study provides evidence for the benefits of concurrent aspirin use with neoadjuvant CRT for rectal cancer, which appears to be independent from tumor *PIK3CA* mutation status, suggesting a broader indication for aspirin in this clinical setting. While compelling, our findings should be considered hypothesis generating and ultimately require additional mechanistic study and prospective clinical validation.

## Figures and Tables

**Table 1:** Patient Demographics.

					Aspirin Use						
					No (*n* = 105)			Yes (*n* = 42)			
Patient Characteristics		Median	*n*	%	Median	*n*	%	Median	*n*	%	*p*
Age, years		61.00			60.00			64.00			0.666
Sex	Male		93	63.3%		60	57.1%		33	78.6%	0.015
	Female		54	36.7%		45	42.9%		9	21.4%	
KPS	80+		139	94.6%		97	92.4%		42	100.0%	0.066
	<80		8	5.4%		8	7.6%		0	0.0%	
Location	0–5 cm		25	17.6%		19	18.8%		6	14.6%	0.491
	5–10 cm		42	29.6%		27	26.7%		15	36.6%	
	10+ cm		75	52.8%		55	54.5%		20	48.8%	
T stage	T1		1	0.7%		1	1.0%		0	0.0%	0.627
	T2		13	9.0%		10	9.6%		3	7.3%	
	T3		123	84.8%		86	82.7%		37	90.2%	
	T4		8	5.5%		7	6.7%		1	2.4%	
N stage	N0		40	28.0%		29	28.7%		11	26.2%	0.118
	N1		65	45.5%		50	49.5%		15	35.7%	
	N2		38	26.6%		22	21.8%		16	38.1%	
Isolated metastatic to liver	No		125	85.0%		91	86.7%		34	81.0%	0.38
	Yes		22	15.0%		14	13.3%		8	19.0%	
Surgery	No		71	48.6%		52	49.5%		20	47.6%	0.73
	Yes		75	51.4%		53	50.5%		22	52.4%	
Induction chemotherapy	No		92	62.6%		63	60.0%		29	69.0%	0.306
	Yes		55	37.4%		42	40.0%		13	31.0%	
Concurrent chemotherapy	Capecitabine		130	88.4%		94	89.5%		36	85.7%	0.306
	5-FU		13	8.8%		7	6.7%		6	14.3%	
	Other		1	0.7%		1	1.0%		0	0.0%	
	None		3	2.0%		3	2.9%		0	0.0%	
Current tobacco use	None		73	50.3%		54	51.9%		19	46.3%	0.475
	Former		46	31.7%		30	28.8%		16	39.0%	
	Current		26	17.9%		20	19.2%		6	14.6%	
Modality	3DCRT		143	97.3%		102	97.1%		41	97.6%	0.873
	VMAT		4	2.7%		3	2.9%		1	2.4%	
Progression	No		86	58.5%		54	51.4%		32	76.2%	0.006
	Yes		61	41.5%		51	48.6%		10	23.8%	
Progression-free survival, months		30.70			23.90			37.32			
Vital status	Alive		103	70.1%		68	64.8%		35	83.3%	0.026
	Dead		44	29.9%		37	35.2%		7	16.7%	
Follow up, months		38.20			34.17			43.03			

**Table 2:** Pathologic treatment response.

Response	No ASA (*n* = 53)	ASA (*n* = 22)
pCR	6 (11.3%)	6 (27.3%)
Partial	29 (54.7%)	9 (40.9%)
No response/progression	18 (34.0%)	7 (31.8%)

Aspirin (ASA); pathologic complete response (pCR).

**Table 3:** Univariate and multivariate Cox regression analysis.

Patient Characteristics	Univariate				Multivariate			
	Progression-Free Survival (PFS)		Overall Survival (OS)		Progression-Free Survival (PFS)		Overall Survival (OS)	
	HR (95% CI)	*p*	HR (95% CI)	*p*	HR (95% CI)	*p*	HR (95% CI)	*p*
Age (<61 years, 61+ years)	0.86 (0.52–1.4)	0.56	1.5 (0.8–2.7)	0.21				
Sex (male, female)	1.1 (0.65–1.8)	0.74	0.93 (0.5–1.7)	0.8				
KPS (80+, <80)	2.1 (0.82–5.2)	0.12	2.5 (0.88–6.9)	0.086			2.3 (0.80–6.72)	0.12
T Stage (T1–2, T3–4)	0.93 (0.44–2)	0.85	1.1 (0.43–2.8)	0.83				
N stage (N0, N+)	0.87 (0.51–1.5)	0.61	0.72 (0.38–1.3)	0.3				
Metastatic to liver	2.6 (1.4–4.7)	0.002	2.3 (1.2–4.4)	0.016	2.59 (1.10–4.79)	0.002	2.10 (1.0–4.2)	0.043
Surgery	0.36 (0.21–0.61)	<0.001	0.2 (0.1–0.41)	<0.001	0.39 (0.23–0.66)	0.001	0.2 (0.10–0.41)	<0.001
Tobacco use	0.98 (0.7–1.4)	0.9	1.2 (0.79–1.7)	0.46				
Induction chemotherapy	1.8 (1.1–3)	0.02	1.5 (0.8–2.7)	0.22	1.33 (0.73–2.24)	0.277		
Beta-blocker use	1.2 (0.68–2.1)	0.52	1.1 (0.57–2.2)	0.76				
Statin use	1.3 (0.69–2.4)	0.44	1.3 (0.63–2.8)	0.45				
Ca channel blocker use	0.68 (0.38–1.2)	0.21	0.67 (0.32–1.4)	0.29				
Angiotensin converting enzyme inhibitors/angiotensin receptor blocker (ACE/ARB) use	0.81 (0.47–1.4)	0.46	1.5 (0.82–2.7)	0.19				
Metformin use	0.66 (0.28–1.5)	0.33	1.1 (0.45–2.5)	0.89				
ASA use	0.4 (0.21–0.8)	0.009	0.42 (0.19–0.93)	0.033	0.35 (0.17–70)	0.003	0.36 (0.16–0.82)	0.015

## Data Availability

Gene expression and cell type analysis data is available in Supplementary Tables S4–S9. The clinical data presented in this study are available on request from the corresponding author. The data are not publicly available due to protected health information.

## Supplementary Materials

The following are available online at https://www.mdpi.com/2072-6694/13/2/205/s1, Figure S1: Consort Diagram, Figure S2: Gene set enrichment analysis, Table S1: Matched Pair balancing, Table S2: Patient characteristics of tumors that underwent RNA sequencing, Table S3: Hotspot mutations, Table S4: Differentially expressed genes by tumor response, Table S5: Differentially expressed genes by aspirin use, Table S6: Gene set enrichment analysis based on tumor response, Table S7: Gene set enrichment analysis based on aspirin use, Table S8: Cell type estimation analysis based on tumor response, Table S9: Cell type estimation analysis based on aspirin use.
