# Supplementary material for: Concurrent Aspirin Use Is Associated with Improved Outcome in Rectal Cancer Patients Who Undergo Chemoradiation Therapy
Source: Cancers (Basel). 2021 Jan 8;13(2):205. doi: 10.3390/cancers13020205 (PMC7826684; doi:10.3390/cancers13020205)
Supplement: Supplementary file 1 [file cancers-13-00205-s001.zip › cancers-1033711-supplementary/revised supp/figures and tables 1,2,3.docx]

Rectal Cancer patients who underwent RT (n=182)

Es

Excluded (n=35)

♦  Palliative intent (n=20)

♦  Adjuvant intent (n=1)

♦  Did not complete treatment (n=9)

♦  Non-adenocarcinoma histology (n=5)

## Chart Review

Rectal Cancer patients who underwent CRT (n=147)

Es

## Analysis

**Figure S1.** Consort Diagram.

**
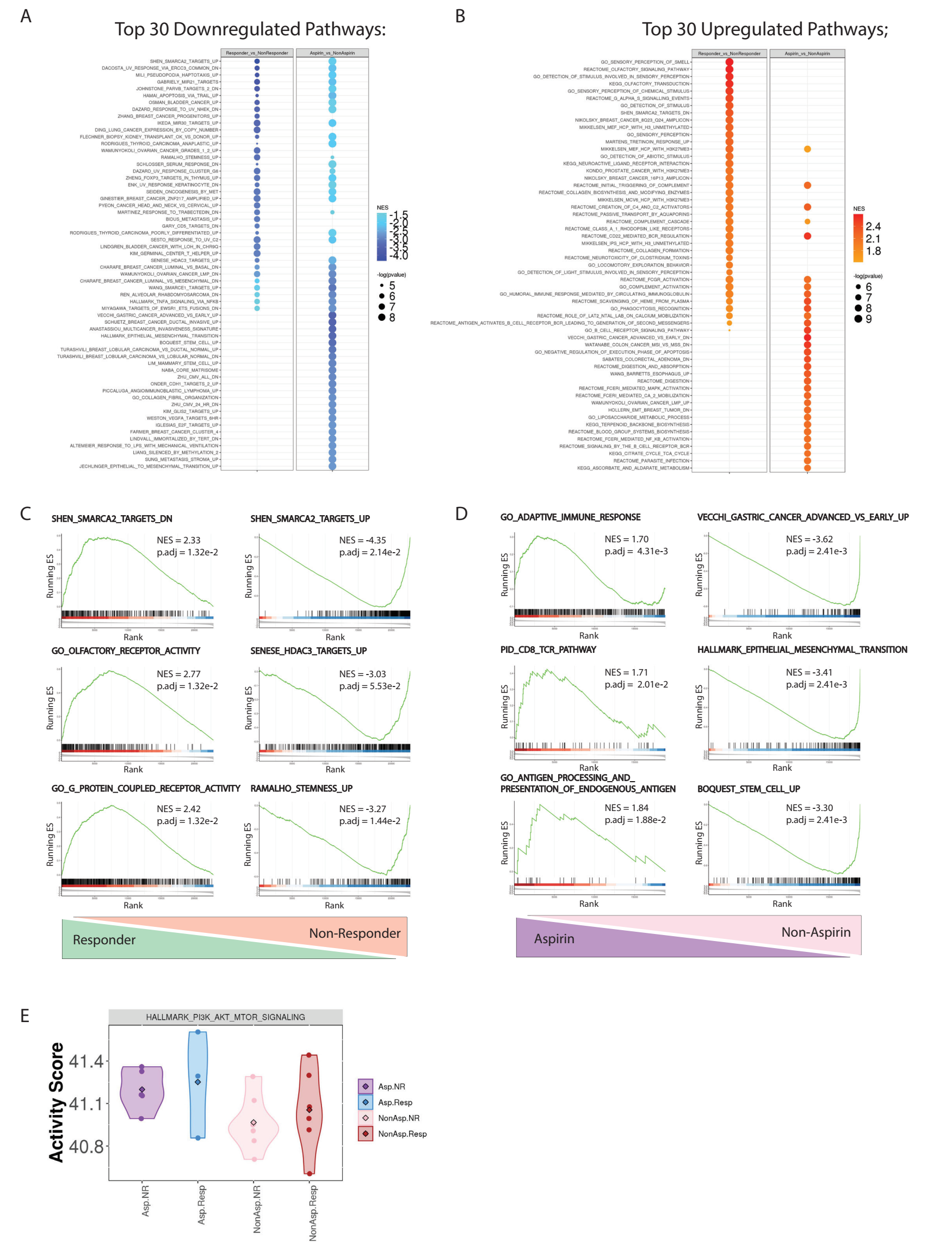
 Figure S2.** Gene set enrichment analysis. (**A**) Top 30 significantly enriched downregulated and (**B**) upregulated pathways between CRT responders and non-responders and aspirin users and non-users determined from GSEA analysis. (**C**) Example enrichment plots for functional pathways significantly associating with CRT response or (**D**) aspirin use. (**E**) Candidate ssGSEA was performed using the HALLMARK_PI3K_AKT_MTOR_SIGNALING pathway. Pathway activity for each sample is shown across groups.

**Table S1.** Matched Pair balancing.

| **Variable** | **p-value** |
| --- | --- |
| Age (<61 years, 61+ years) | 0.81 |
| Sex (male, female) | 0.78 |
| KPS (80+, <80) | 1 |
| T Stage (T1-2, T3-4) | 0.67 |
| N stage (N0, N+) | 1 |
| Metastatic to liver | 1 |
| Induction chemotherapy | 1 |
| Surgery | 1 |

**Table S2.** Patient characteristics of tumors that underwent RNA sequencing.

|  | | **Aspirin use** | | | | | |  |
| --- | --- | --- | --- | --- | --- | --- | --- | --- |
|  |  | No (n=13) | | | Yes (n=8) | | |  |
|  |  | Median [IQR] | n | % | Median [IQR] | n | % | p-value |
| **Age (years)** | | 58 [54-65] |  |  | 60.5 [51-74] |  |  | 0.228 |
| **KPS** | 70 |  | 2 | 15.4% |  | 0 | 0.0% | 0.451 |
|  | 80 |  | 8 | 61.5% |  | 5 | 62.5% |  |
|  | 90 |  | 3 | 23.1% |  | 3 | 37.5% |  |
| **Location** | 0-5 cm |  | 3 | 23.1% |  | 1 | 12.5% | 0.83 |
|  | 5-10 cm |  | 4 | 30.8% |  | 3 | 37.5% |  |
|  | 10+ cm |  | 6 | 46.2% |  | 4 | 50.0% |  |
| **T stage** | T1 |  | 0 | 0.0% |  | 0 | 0.0% | 0.507 |
|  | T2 |  | 1 | 7.7% |  | 0 | 0.0% |  |
|  | T3 |  | 11 | 84.6% |  | 8 | 100.0% |  |
|  | T4 |  | 1 | 7.7% |  | 0 | 0.0% |  |
| **N stage** | N0 |  | 1 | 7.7% |  | 3 | 37.5% | 0.062 |
|  | N1 |  | 8 | 61.5% |  | 1 | 12.5% |  |
|  | N2 |  | 4 | 30.8% |  | 4 | 50.0% |  |
| **Concurrent chemo** | Capecitabine |  | 13 | 100.0% |  | 6 | 75.0% | 0.058 |
|  | 5-FU |  | 0 | 0.0% |  | 2 | 25.0% |  |
| **Current tobacco use** | None |  | 5 | 38.5% |  | 4 | 50.0% | 0.633 |
|  | Former |  | 4 | 30.8% |  | 3 | 37.5% |  |
|  | Current |  | 4 | 30.8% |  | 1 | 12.5% |  |
| **Tumor response** | PR |  | 6 | 46.2% |  | 3 | 37.5% | 0.697 |
|  | No response/progression |  | 7 | 53.8% |  | 5 | 62.5% |  |

**Table S3.** Hotspot mutations.

| **Gene** | **Mutation** | **Response to chemoradiation therapy** | **Aspirin** |
| --- | --- | --- | --- |
| PIK3CA | E545D | Partial | no |
| KRAS | G13D | No response, progression, or nodal downstage | yes |
| KRAS | G12D | No response, progression, or nodal downstage | no |
